# Recordings in an integrating central neuron reveal the mode of action of isoeugenol

**DOI:** 10.1038/s42003-023-04695-4

**Published:** 2023-03-23

**Authors:** Peter Machnik, Nastaran Biazar, Stefan Schuster

**Affiliations:** Department of Animal Physiology, University of Bayreuth, (Universitätsstraße 30, D-95440 Bayreuth), Bayreuth, Germany

**Keywords:** Target identification, Sensory processing, Ethics

## Abstract

Although isoeugenol is one of the most widely used anesthetics in fish, its actual mode of action and thus its applicability for particular interventions is poorly understood. Here we determined effects of isoeugenol on various aspects of sensory and neural function, taking advantage of intracellular in vivo recordings in a uniquely suited identified neuron, the Mauthner neuron in the brain of goldfish. We show that isoeugenol strongly affects hearing and vision, but sensitivity and time course of action differed largely in these two senses. The action potential, chemical and electric synaptic transmission at the central neuron were not affected at low but efficient anesthesia. Effects seen at high concentration thereby do not support current views of how isoeugenol might act on central neurons. We show that isoeugenol is highly useful to anesthetize fish for handling, but that in more severe treatment its application needs to be carefully adapted to task.

## Abstract (web summary)

In vivo intracellular recordings from the goldfish Mauthner neuron reveal the mode of action of isoeugenol, which is a widely used anaesthetic in fish.

## Introduction

The need for efficient anesthetics in fish arises not only in laboratories but to a considerable extent also in aquaculture and fish farming^1–5^. With growing concern that fish are sentient animals, animal welfare and ethical implications of handling and processing fish are becoming important^4–15^. Many countries now require appropriate use of anesthetics for all vertebrates, including fish, for any procedure that could be painful or stressful^4,16,17^. This creates a challenging situation: In principle, several anesthetics are available for use in fish and some of them are used extensively, but often with different traditions in aquaculture and in research^15,18,19^. In both, however, evidence is largely missing about their mode of action, the required concentrations, the required time of exposure and time till full recovery and it is often not known how anesthetic agents affect the functions that should be studied in the anesthetized animal^20–22^. In contrast to mammals – for which many more studies are available – such information is presently mostly lacking for fish and is urgently needed^18,22^.

**Fig. 1:**
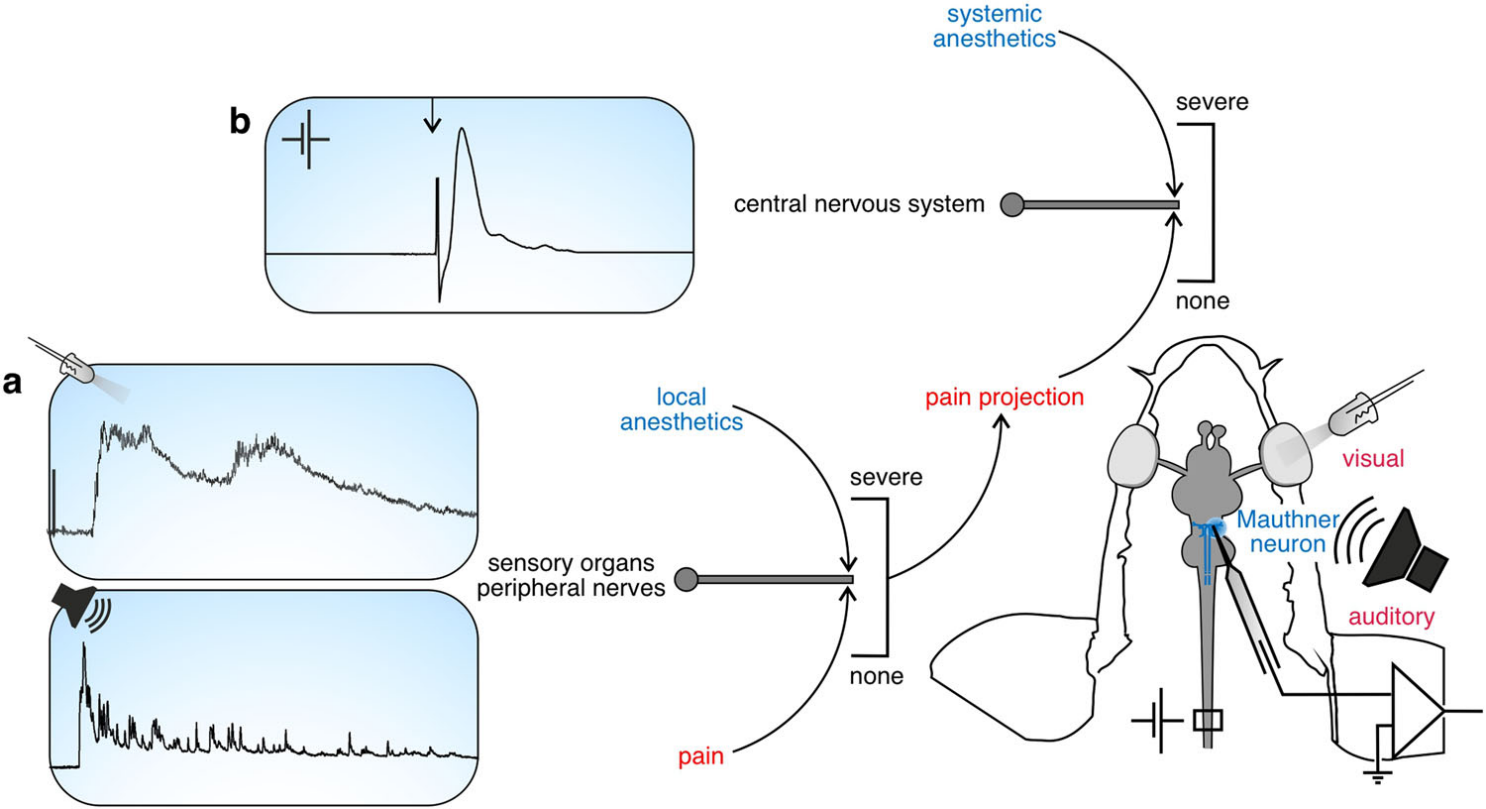
Brief overview of how the Mauthner neuron can be used to examine the mode of action of anesthetics. Intracellular in vivo recording in the Mauthner neuron (MN) of the goldfish provides a comprehensive and rapid assay of targets an anesthetic can act on. **a** Its function as a command neuron for life-saving escapes requires the MN to faithfully process information forwarded from almost all senses. So, recording of postsynaptic potentials after sensory stimulation (e.g., visual or acoustic) can reveal whether the anesthetic affects different sensory organs directly, the transmission of sensory information to the MN or the processing within the MN. **b** The systemic effect an anesthetic can have on central neurons by affecting ion channels and the generation and axonal conduction of action potentials can be studied by antidromically activating the Mauthner axon.

One of the most widely used anesthetics employed in fish is isoeugenol^19^. Although it is used in vast quantities each year in aquaculture, neither its mode of action, critical concentration, effects on senses and central processing during long-term exposure are known and it is not clear whether it might also be useful, outside aquaculture, in research laboratories. Specifically, it is still unclear whether isoeugenol acts as a local anesthetic^18,23,24^ – i.e., prevents sensory information from reaching the brain^20^ – or inhibits sodium channels of neurons in the brain^25^ and thus acts as a systemic anesthetic^20,22^. Here we use an approach that we introduced recently^21,26,27^ to examine the mode of action of isoeugenol and to provide evidence critical for using it in research labs (Fig. 1). By recording intracellularly in an identified neuron in the brain of fish whose natural function requires it to reliably receive and process information from all senses^27–29^ it is possible to not only characterize the effects of agents on the action potential and chemical and electrical transmission in a central neuron, but also on various senses and their transmission to the central nervous system (CNS).

To study the anesthetic effects a substance can have in fish and to narrow down its mode of action, the Mauthner neuron (MN) thereby is an ideal substrate. The MN is one of the very few neurons in the vertebrate brain that can be identified individually from one animal to the next and that is readily accessible to intracellular in vivo recording^21,26,30^. It has therefore been a major source of insight into fundamental mechanisms of synaptic communication in the vertebrate CNS^31^. Playing a key role in a vital escape response network^29^, the MNs are present in most teleost fish species^28^ and all available evidence suggests that the morphological and physiological properties of the teleost MN are phylogenetically conserved^32,33^. Its natural function requires the MN to integrate information from various sensory systems. The MN responds to all information taken up from the environment, i.e., gets synaptic input from senses processing mechanosensory, somatosensory, trigeminal, visual information. In fish having further senses information taken up by these senses is also forwarded to the MN. Electric fish, for instance, in addition feed information from their electroreceptors into the MN^34^. This situation might be so because a priori the nervous system cannot know which stimuli signal danger and at which level. Adding all sensory information also allows the animal to respond to hints that regularly are associated with danger. A typical teleost MN has two major dendrites^32^. In the goldfish, the ventral dendrite integrates and processes somatosensory information^35^ and information forwarded from the eyes^36,37^, whereas the lateral dendrite integrates and processes mechanosensory information forwarded from auditory hair cells and lateral line^38,39^. It is generally agreed that this allocation of sensory information processing is conserved in teleost fish^28,33,37,40^.

What can be learned by studying just one neuron within the brain of a fish? Here it is important to recall the context in which this neuron operates. As a true command neuron that drives life-saving escape responses^29^, the MN integrates information from diverse sensory systems and thereby relies on preprocessing in different sensory areas, functioning of the sensory organs and their transmission toward the MN in the CNS. By probing the MN, it is thus possible to obtain information on diverse aspects of brain and sensory function. Applying different sensory stimuli in vivo allows to work out the effect that the anesthetic has on each given sensory channel, from sensory transduction, the transmission of this information to the CNS and central processing^21,27^. By stimulating the axon antidromically, the effect of an anesthetic on action potential generation and spreading in the CNS can be studied,which allows to test hypotheses of whether an anesthetic acts generally on axons in the CNS. Moreover, all that is needed to obtain the various pieces of evidence is to switch from one stimulus to another, i.e., activating the MN, giving visual stimuli, and giving acoustic stimuli, and so on. While stimuli are switched, the intracellular recording always remains in place and simply records the various responses. All these aspects make the MN preparation so extremely efficient, with a large variety of crucial aspects determined in just 2 h of experimentation.

Using this approach, we show here that isoeugenol does act as a potent local anesthetic, but that it affects different sensory systems very differently. At high concentrations it can additionally have a systemic effect that is, however, unlikely to be caused by the inhibition of sodium channels. The detailed information we provide here thereby suggests that isoeugenol is useful in many tasks in research and aquaculture, for example, to reduce stress during handling or other stressful procedures, to provide immobilization during imaging, and to reduce the response when using bright lights during an external examination.

## Results and Discussion

### Surgical anesthesia is reached in goldfish by applying 10 mg L^−^^1^ isoeugenol

Generally, the depth (stage) of anesthesia is determined in fish by evaluating whether equilibrium, respiratory rate, muscle tone, and behavioral responses of the fish are affected. In the present study, we defined depth of anesthesia referring to Ross and Ross (2008)^41^ and Stoskopf and Posner (2008)^42^. Stage III.2 anesthesia thereby is needed to be reached in fish, when surgical intervention needs to be performed. It involves total loss of equilibrium, pain perception and observable responsiveness to tactile stimuli. In contrast, during stage III.1 anesthesia the fish still respond to strong tactile stimuli, whereas stage IV anesthesia corresponds to a medullary collapse – an irreversible loss of central neuron function in the hindbrain, finally leading to death. In the present study, all experimental fish exposed to isoeugenol at the concentration of 10 mg L^−^^1^ (*n* = 40 (of 40) fish) lost equilibrium and ceased swimming within 10 min. They were lying on the ground but still breathing. None of them showed any response to touch when grabbed or pressure was exerted on the fish’s caudal peduncle. In responsive fish, this kind of stimuli triggers an escape response and subsequent swimming behavior. After 15 min of exposure to the anesthetic the fish were placed in the electrophysiological recording chamber. Artificial respiration was established and surgery started (see Methods). During surgical intervention none of the fish showed any response, indicating the effectiveness of the stage III.2 anesthesia as established by isoeugenol at the concentration of 10 mg L^−^^1^. As seen from MN recording (next paragraph), stage IV anesthesia was not reached by isoeugenol anesthesia even at concentrations as high as 60 mg L^−^^1^, indicating a high safety margin of isoeugenol anesthesia. However, as also seen from MN recording, isoeugenol anesthesia is not systemic and does not impair all senses.

### Isoeugenol affects the action potential of a central neuron only at high concentration and after long exposure

**Fig. 2:**
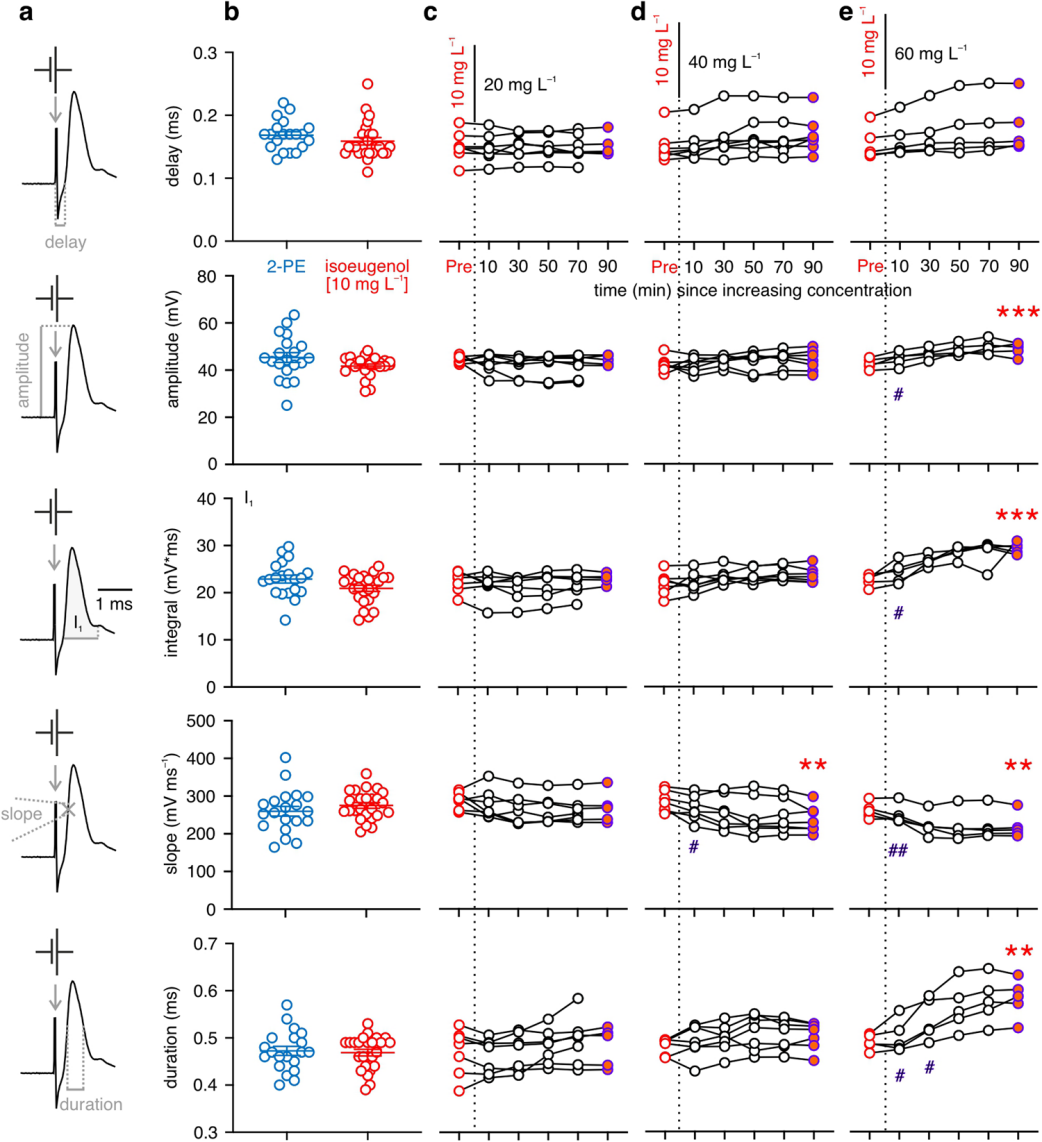
Isoeugenol affects the action potential of a central neuron only at high concentration. **a** Example of an action potential (AP) recorded in the Mauthner neuron (MN) to illustrate the measurements taken. **b** In fish anesthetized with isoeugenol at the concentration of 10 mg L^−^^1^ (*n* = 25; red circles indicate individual fish) no effect was seen on the AP, i.e., all values were not significantly different from those determined in control fish anesthetized with 2-PE [400 mg L^−^^1^] (*n* = 20; blue circles). Means ± standard errors of mean are indicated. **c**–**e** Dotted vertical lines indicate moment of change to higher concentration at time = 0 min. Connected circles indicate mean values in individuals. **c** No significant changes after increase in isoeugenol concentration from 10 to 20 mg L^−^^1^ (*n* = 7 fish). **d**, **e** At 40 (*n* = 7 fish) and 60 mg L^−^^1^ (*n* = 5 fish), the slope of the AP decreased slightly within 10–30 min after increasing dose. At 60 mg L^−^^1^, peak amplitude, I_1_ and the duration of the AP were also affected. Effects also took more than 10 min to occur. Red asterisks highlight significant differences between values at baseline concentration (Pre; red circles) and values obtained 90 min after the increase in concentration (orange-filled circles). Violet hashtags indicate significant differences between values obtained 90 min after dose increase (orange-filled circles) and values determined also after dose increase but earlier (black-outlined circles). One, two, three symbols indicate *P* ≤ 0.05, *P* ≤ 0.01, or *P* ≤ 0.001, respectively.

All experiments started at an isoeugenol concentration of 10 mg L^−^^1^ to which the fish had already been exposed for 45 to 60 min during the preparation stages for the intracellular recording (see Methods). In the subsequent experiments the concentration was then either left at 10 mg L^−^^1^ (control) or was increased to either 20, 40, or 60 mg L^−^^1^ (Supplementary Fig. 1). Additionally, we ran controls in which fish were anesthetized with 2-phenoxyethanol (2-PE) at a concentration of 400 mg L^−^^1^, where no aspect of the action potential (AP) or of the acoustically or visually induced postsynaptic potential in the MN is affected^26^. To assay the effects of isoeugenol on the AP and its conduction we electrically stimulated the spinal cord to antidromically elicit APs in the MN. This allowed us to characterize the delay between electrical stimulation of the spinal cord and the rise of the AP in the MN soma, peak amplitude and half-maximal duration of the AP, its maximal slope as well as the area under the AP in the first millisecond (Fig. 2a). Surprisingly, isoeugenol at a concentration of 10 mg L^−^^1^ for more than an hour had no significant effect on any of these values. Neither the delay nor any other of the properties determined for the AP were significantly different from values determined in 2-PE controls (Fig. 2b; Table 1). The same held true even in fish that had faced additional 90 min in the increased concentration of 20 mg L^−^^1^ (Fig. 2c; Table 1). In fish that faced a concentration of 40 mg L^−^^1^ the slope of the AP was the only aspect that was affected (Fig. 2d; Table 1). It decreased from 284.9 ± 10.5 mV ms^−^^1^ to 238.2 ± 13.4 mV ms^−^^1^. However, this effect occurred only after 10 to 30 min of exposure to the higher concentration (mixed-effects model: *F* = 10.32, *P* = 0.0009; Dunnett test: [90 min vs. *Pre*, 10 min]: *P* ≤ 0.0216; [90 min vs. 30–70 min]: *P* ≥ 0.2253). Additional effects on the AP were seen only in fish that encountered the largest increase in dose, from 10 to 60 mg L^−^^1^ (Fig. 2e; Table 1). Peak amplitude of the AP was increased by 15% from 42.48 ± 0.96 mV to 48.75 ± 1.26 mV, I_1_ was increased by 30% from 22.65 ± 0.55 mV*ms to 29.50 ± 0.52 mV*ms. The duration of the AP was increased from 0.49 ± 0.01 ms to 0.58 ± 0.02 ms. Maximal slope was also decreased. Again, none of these effects occurred quickly, but took at least 10–30 min to establish (amplitude, I_1_, maximal slope: mixed-effects model: *F* ≥ 16.36, *P* ≤ 0.0039; Dunnett test: [90 min vs. *Pre*, 10 min]: *P* ≤ 0.0312; [90 min vs. 30 to 70 min]: *P* ≥ 0.0876). The duration of the AP changed only after 30–50 min (mixed-effects model: *F* = 21.23, *P* = 0.0012; Dunnett test: [90 min vs. *Pre*, 10 to 30 min]: *P* ≤ 0.0215; [90 min vs. 50–70 min]: *P* ≥ 0.2820). Hence, 10 mg L^−^^1^ of isoeugenol, a dose that was perfectly sufficient to anesthetize the experimental fish, did not exert a clear effect on the AP. An effect is seen here only at much higher concentrations and after prolonged exposure. Furthermore, our finding of an increase in AP amplitude is not easily reconciled with the view that isoeugenol generally acts by blocking voltage-dependent sodium channels^22,23^.

**Table 1:** Isoeugenol affects the action potential of the Mauthner neuron only at high concentration.

	2-PE (400 mg L^−^^1^) (*n* = 20; 44 ≤ mpf ≤ 64) vs. isoeugenol (10 mg L^−^^1^) (*n* = 25; 44 ≤ mpf ≤ 91)		direction of sign. diff.
Delay	Mann-Whitney test	*P* = 0.1092	
Amplitude	Mann-Whitney test	*P* = 0.0647	
Area I_1_	Unpaired t-test	*P* = 0.0656; *t* = 1.889	
Slope	Unpaired t-test	*P* = 0.2891; *t* = 1.073	
Duration	Unpaired t-test	*P* = 0.7538; *t* = 0.316	

	isoeugenol (10 mg L^−^^1^) (*n* = 5; 44 ≤ mpf ≤ 72) vs. isoeugenol (20 mg L^−^^1^) (53 ≤ mpf ≤ 72)		direction of sign. diff.
Delay	Paired t-test	*P* > 0.999; *t* < 0.001	
Amplitude	Paired t-test	*P* = 0.8469; *t* = 0.206	
Area I_1_	Paired t-test	*P* = 0.7416; *t* = 0.354	
Slope	Paired t-test	*P* = 0.1786; *t* = 1.629	
Duration	Paired t-test	*P* = 0.8149; *t* = 0.250	

	isoeugenol (10 mg L^−^^1^) (*n* = 7; 48 ≤ mpf ≤ 91) vs. isoeugenol (40 mg L^−^^1^) (50 ≤ mpf ≤ 92)		direction of sign. diff.
Delay	Wilcoxon test	*P* = 0.0625	
Amplitude	Paired t-test	*P* = 0.4904; *t* = 0.735	
Area I_1_	Paired t-test	*P* = 0.0671; *t* = 2.232	
Slope	Paired t-test	***P*** = **0.0015**; *t* = 5.528	iso (10 mg) > iso (40 mg)
Duration	Wilcoxon test	*P* = 0.0781	

	isoeugenol (10 mg L^−^^1^) (*n* = 5; 45 ≤ mpf ≤ 85) vs. isoeugenol (60 mg L^−^^1^) (52 ≤ mpf ≤ 76)		direction of sign. diff.
Delay	Wilcoxon test	*P* = 0.0625	
Amplitude	Paired t-test	***P*** = **0.0007**; *t* = 9.607	iso (10 mg) < iso (60 mg)
Area I_1_	Paired t-test	***P*** = **0.0003**; *t* = 11.560	iso (10 mg) < iso (60 mg)
Slope	Paired t-test	***P*** = **0.0059**; *t* = 5.345	iso (10 mg) > iso (60 mg)
Duration	Paired t-test	***P*** = **0.0023**; *t* = 6.903	iso (10 mg) < iso (60 mg)

*iso* Isoeugenol, *mpf* Measurement repetitions per fish, *sign. diff.* Significant difference; for significant differences *P*-values are highlighted (in bold); *n* indicates the number of independent animal samples.

### Isoeugenol affects acoustic inputs even at low concentration

**Fig. 3:**
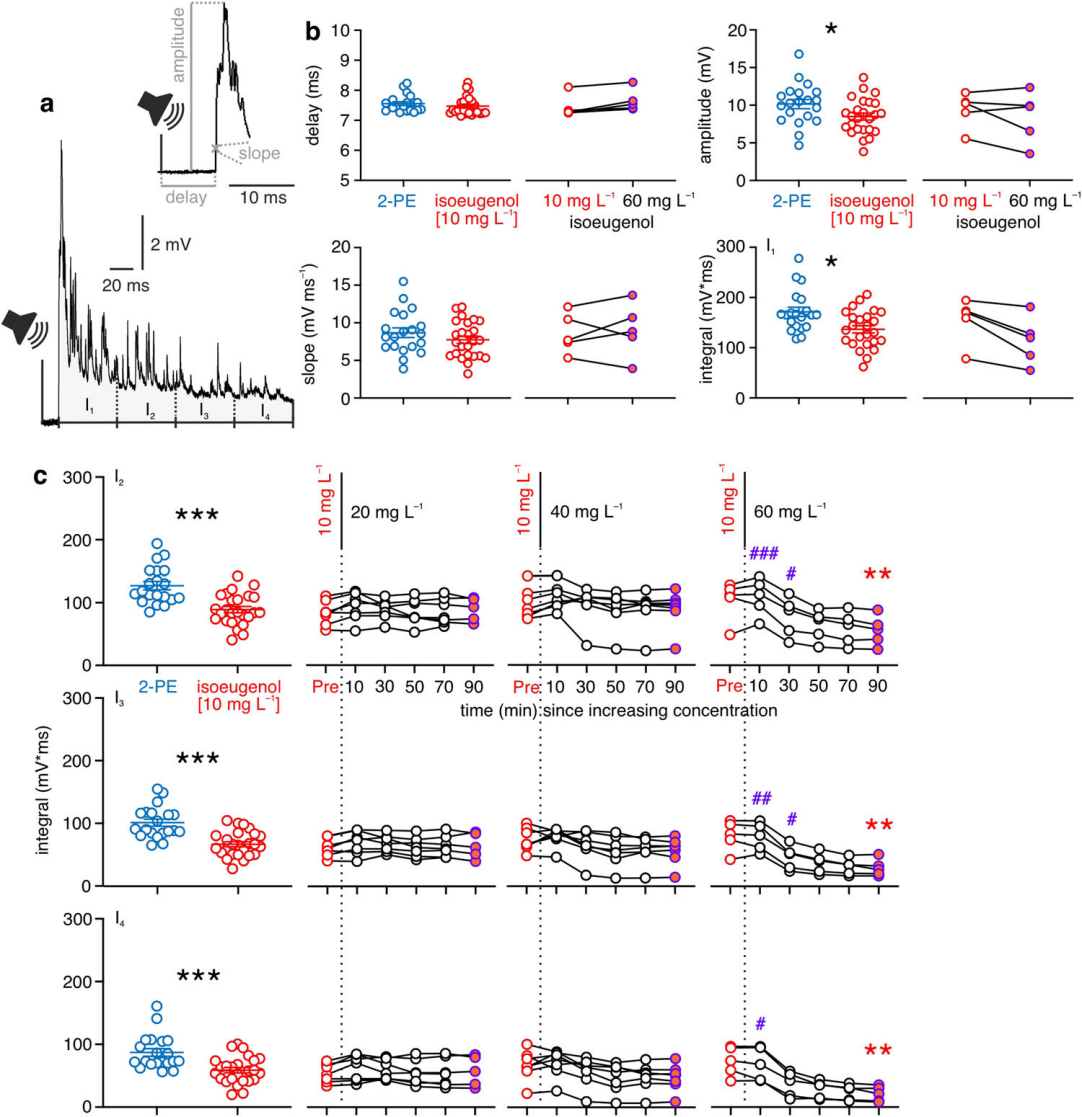
Isoeugenol slightly affects acoustic inputs into the Mauthner neuron. **a** Exemplary PSP recorded after acoustic stimulation of the fish, with indication of measurements taken. **b** Acoustic PSPs were slightly reduced in peak amplitude and in I_1_ in fish anesthetized with isoeugenol [10 mg L^−^^1^] (*n* = 25; red circles for individual fish), compared to the 2-PE controls (*n* = 20; blue circles). Means ± standard errors of mean are indicated. Slope and delay were not affected. This remained so at isoeugenol concentrations up to 60 mg L^−^^1^ (for time course see Supplementary Fig. 2). Changes of values obtained at 10 mg L^−^^1^ and 90 min after increasing dose in the *n* = 5 experimental fish, in which the concentration was increased from 10 to 60 mg L^−^^1^, indicated on the right for each individual fish. **c** Detailed analysis of integrals I_2_ to I_4_, arranged as in Fig. 2 with time indicating exposure time passed since switch (dotted vertical line at time = 0 min) to higher concentration. Significant differences between values obtained before the increase in isoeugenol concentration (Pre; red circles) and values obtained 90 min after the increase in concentration (orange filled circles) are indicated in (**c**) by red asterisks. Significant differences between values obtained 90 min after dose increase (orange filled circles) and values determined also after dose increase but earlier (black outlined circles) are indicated by violet hashtags. One, two, and three symbols indicate *P* ≤ 0.05, *P* ≤ 0.01, or *P* ≤ 0.001, respectively. Same fish as in Fig. 2. Connected circles indicate mean values of individual fish.

In each experimental fish we could – simply by switching from antidromic to sensory stimulation – also characterize the effects of the concentrations introduced above on postsynaptic potentials (PSPs) elicited by either acoustic or visual stimuli. The evidence reported above suggests that 10 mg L^−^^1^ isoeugenol does not act on the AP of the MN. Since the MN does not differ from most other central neurons in the way it generates and conducts APs, we assume that the clear anesthetic effect of isoeugenol [10 mg L^−^^1^] would not be due to its effect on central neuron function, but due to blocking peripheral inputs to the CNS (Fig. 1a). To examine the effect of isoeugenol on such inputs, we applied standardized brief acoustic pulses or light stimuli (next paragraph) and determined the following aspects of the induced PSPs: the delay between onset of the stimulus and onset of the PSP, the maximal amplitude and slope of the PSP as well as the area under the PSP in four consecutive integrals (I_1_ to I_4_) (Fig. 3a). To account for their different durations the intervals we considered were 50 ms for the acoustic and 75 ms for the visual PSPs. Figure 3 reports first the effects on the acoustic PSPs. Strikingly, 10 mg L^−^^1^ of isoeugenol did have significant effects on the acoustic inputs to the MN (Fig. 3b, c; Table 2). Compared to the 2-PE controls, isoeugenol [10 mg L^−^^1^] significantly reduced the peak as well as the overall amplitude of the acoustically induced PSPs. At 10 mg L^−^^1^ isoeugenol had no significant effect on PSP delay and maximal slope. For fish that faced an increase in isoeugenol concentration from 10 to values up to 60 mg L^−^^1^ for additional 90 min some effects did not increase any further: Peak amplitude and I_1_ remained unchanged (Fig. 3b; Table 2; for time course see Supplementary Fig. 2), but I_2_ to I_4_ decreased at the highest concentration (Fig. 3c) after 10 min (I_4_; mixed-effects model: *F* ≥ 44.27, *P* ≤ 0.0008; Dunnett test: [90 min vs. *Pre*, 10 min]: *P* ≤ 0.0135; [90 min vs. 30, 70 min]: *P* ≥ 0.0912)) or more than 30 min (I_2_, I_3_) (mixed-effects model: *F* ≥ 61.09, *P* ≤ 0.0003; Dunnett test: [90 min vs. *Pre*, 10–30 min]: *P* ≤ 0.0034; [90 min vs. 50–70 min]: *P* ≥ 0.0978). Delay and maximal slope of the acoustically induced PSP were, however, still not affected even at 60 mg L^−^^1^ isoeugenol (Fig. 3b; Table 2).

**Table 2:** Effects of isoeugenol anesthesia on the acoustically in the Mauthner neuron-induced postsynaptic potential.

	2-PE (400 mg L^−^^1^) (*n* = 20; 14 ≤ mpf ≤ 43) vs. isoeugenol (10 mg L^−^^1^) (*n* = 25; 17 ≤ mpf ≤ 27)		direction of sign. diff.
Delay	Mann-Whitney test	*P* = 0.1251	
Amplitude	Mann-Whitney test	***P*** = **0.0312**	2-PE > iso (10 mg)
I_1_	Mann-Whitney test	***P*** = **0.0114**	2-PE > iso (10 mg)
I_2_	Unpaired t test	***P*** < **0.0001**; *t* = 4.691	2-PE > iso (10 mg)
I_3_	Unpaired t test	***P*** < **0.0001**; *t* = 5.140	2-PE > iso (10 mg)
I_4_	Mann-Whitney test	***P*** = **0.0005**	2-PE > iso (10 mg)
Slope	Unpaired t test	*P* = 0.2386; *t* = 1.195	

	isoeugenol (10 mg L^−^^1^) (*n* = 5; 14 ≤ mpf ≤ 40) vs. isoeugenol (60 mg L^−^^1^) (17 ≤ mpf ≤ 25)		direction of sign. diff.
Delay	Wilcoxon test	*P* = 0.0625	
Amplitude	Paired t test	*P* = 0.3303; *t* = 1.107	
I_1_	Wilcoxon test	*P* = 0.0625	
I_2_	Paired t test	***P*** = **0.0031**; *t* = 6.387	iso (10 mg) > iso (60 mg)
I_3_	Paired t test	***P*** = **0.0022**; *t* = 6.999	iso (10 mg) > iso (60 mg)
I_4_	Paired t test	***P*** = **0.0019**; *t* = 7.309	iso (10 mg) > iso (60 mg)
Slope	Paired t test	*P* = 0.6779; *t* = 0.447	

*iso* Isoeugenol, *mpf* Measurement repetitions per fish, *sign. diff.* Significant difference; for significant differences *P*-values are highlighted (in bold), *n* indicates the number of independent animal samples.

### Isoeugenol affects visual inputs even stronger than acoustic ones

**Fig. 4:**
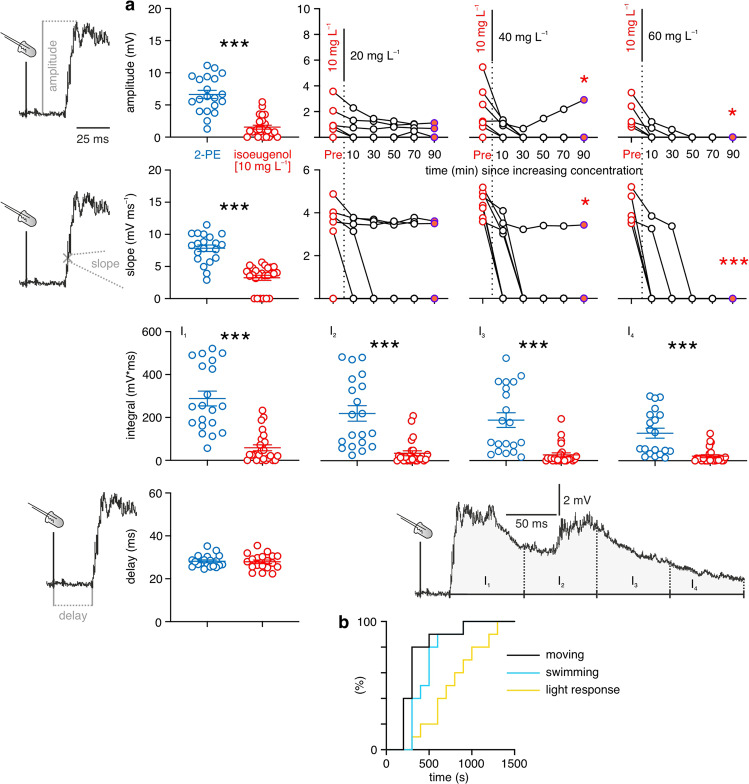
Isoeugenol massively affects visual inputs into the Mauthner neuron. **a** Anesthetizing fish with 10 mg L^−^^1^ isoeugenol affected all aspects of the visual PSP (*n* = 25; red circles indicate individual fish), as seen by comparison with 2-PE anesthetized control fish (*n* = 20; blue circles), except delay. Measured parameters (amplitude, slope, integrals, delay) are illustrated. Means ± standard errors of mean are indicated. PSP peak amplitude and slope were reduced further when isoeugenol concentration was increased to 20, 40, or 60 mg L^−^^1^. Note absence of detectable visual PSPs in many fish (individual circles, connected by lines). Diagrams and fish as in Fig. 2. Significant differences between values obtained before the increase in isoeugenol concentration (Pre; red circles) and values obtained 90 min after the increase in concentration (orange filled circles) are indicated by red asterisks. One, two, three asterisks indicate *P* ≤ 0.05, *P* ≤ 0.01 or *P* ≤ 0.001, respectively. Connected circles indicate mean values of individual fish. **b** Recovery of vision in 10 fish that had been anesthetized with 20 mg L^−^^1^ isoeugenol for 30 min. Percentage of fish showing the three indicated behaviors after absence (at time = 0 min) of anesthetic.

Surprisingly the lowest concentration of isoeugenol (10 mg L^−^^1^) anesthesia affected the visual inputs to the MN to a greater degree than acoustic inputs, reducing the PSPs not by 20% (acoustic PSPs, Fig. 3b), but by about 75%, from 6.64 ± 0.63 mV in 2-PE fish to 1.55 ± 0.31 mV in fish anesthetized with isoeugenol (Fig. 4a; Table 3). Also the areas I_1_ to I_4_ were all reduced. Additionally, the maximal slopes of the PSPs were reduced by approximately 60% from 7.84 ± 0.51 mV ms^−^^1^ in 2-PE animals to 3.24 ± 0.39 mV ms^−^^1^ in fish anesthetized with isoeugenol. The only aspect that was not affected in comparison to 2-PE controls was the delay between onset of the light flash and onset of the PSP. For the visual PSPs increasing the dose of isoeugenol (to 20, 40, or 60 mg L^−^^1^) still increased the already strong effect. At 20 mg L^−^^1^ PSP amplitudes decreased to 0.62 ± 0.34 mV after 10 min (*n* = 7 independent animal samples; measurement repetitions per fish: 8–40), 0.48 ± 0.24 mV after 30 min (measurement repetitions per fish: 8–40), and 0.30 ± 0.20 mV after 90 min (measurement repetitions per fish: 9–44) of exposure. At the highest concentration (60 mg L^−^^1^) the visual stimuli (but not the acoustic stimuli) failed to induce a PSP in the MN after 50 min (*n* = 5 independent animal samples): PSP peak amplitude was 0.38 ± 0.25 mV after 10 min (measurement repetitions per fish: 5–40), 0.12 ± 0.12 mV after 30 min (measurement repetitions per fish: 5–40), and 0.00 mV after 50 min of exposure. It is important to stress that the effects we find after long exposure are demonstrably not due to time effects, i.e., a general decline in the state of the preparation. This is seen in the control group (Supplementary Fig. 1a) that remained at 10 mg L^−^^1^ and showed no trend in any aspects of the sensory PSPs and the APs (Supplementary Fig. 3) and no effect of isoeugenol [10 mg L^−^^1^] anesthesia occurring late, after more than 60 min of exposure (mixed-effects model: *F* ≤ 3.966, *P* ≥ 0.1631). Moreover, we also stress that our experimental design ensured that all fish faced all types of stimuli at all times of the exposure (Supplementary Fig. 1 and Figs. 2–4). Hence, our findings show that isoeugenol clearly affects the inputs to the MN much stronger than the MN itself, which would be compatible with a peripheral mode of action of isoeugenol. However, the differential effect of isoeugenol on visual and acoustic inputs strongly suggests that the effect is not due to a general blockage of sensory axons that connect the periphery with the MN and other neurons in the CNS. In such a general scenario, the effects of increased doses should have acted similarly (i.e., with similar time course and dose-dependency) on both visual and acoustic inputs. The distinctly different sensitivity we found thus suggests that isoeugenol might at least have an additional strong effect on the sensory organs themselves, different in vision and hearing. This conclusion is also supported by findings in an individual fish in which the time course of action was demonstrably reversed between the visual and acoustic stimuli (Supplementary Fig. 4).

**Table 3:** Effects of isoeugenol anesthesia on visual input to the Mauthner neuron.

	2-PE (400 mg L^−^^1^) (*n* = 20; 13 ≤ mpf ≤ 41) vs. isoeugenol (10 mg L^−^^1^) (*n* = 25; 5 ≤ mpf ≤ 44)		direction of sign. diff.
Delay	Unpaired t-test	*P* = 0.8132; t = 0.2380	
Amplitude	Mann-Whitney test	***P*** < **0.0001**	2-PE > iso (10 mg)
I_1_	Mann-Whitney test	***P*** < **0.0001**	2-PE > iso (10 mg)
I_2_	Mann-Whitney test	***P*** < **0.0001**	2-PE > iso (10 mg)
I_3_	Mann-Whitney test	***P*** < **0.0001**	2-PE > iso (10 mg)
I_4_	Mann-Whitney test	***P*** < **0.0001**	2-PE > iso (10 mg)
Slope	Unpaired t test	***P*** < **0.0001**; *t* = 7.312	2-PE > iso (10 mg)

*iso* Isoeugenol, *mpf* Measurement repetitions per fish, *sign. diff.* Significant difference, for significant differences *P*-values are highlighted (in bold), *n* indicates the number of independent animal samples.

### Reversibility of the effects

Our findings reveal that isoeugenol acts most strongly on vision, and most likely on its peripheral aspects. Furthermore, our findings also suggest that isoeugenol causes long-lasting effects, and can even cause a complete blockade of visual inputs into the CNS. We therefore examined the possibility that vision would be affected long after anesthetization with isoeugenol, which might largely restrict its usefulness. To test this, we exposed fish to an isoeugenol concentration of 20 mg L^−^^1^ for 30 min, then placed them in fresh water with no anesthetic agent and monitored the recovery of swimming behavior and of the responsiveness to light stimuli. Control fish (without isoeugenol) immediately responded to standardized light flashes (same as to elicit PSPs). 9 of 10 control fish turned in response to the first light flash, the remaining tenth fish turned after the second light flash. Isoeugenol [20 mg L^−^^1^] fish were initially solely breathing after transfer to the recovery tank, but none was moving during the first 3 min of recovery. The first fish moved after 4 min and all fish were swimming after 15 min of recovery. At this time already seven (of 10) fish readily responded to light flashes and after 22 min of recovery all fish responded to light flashes. Hence, despite the strong effect isoeugenol at 20 mg L^−^^1^ had on vision (Fig. 4a), the effect seems reversible and overcome after at least about 15 min (Fig. 4b).

### Conclusion: Isoeugenol acts more as a local, not systemic anesthetic

Our recordings in a central multisensory neuron that is essential for the life-saving rapid startle response^29^ reveal that isoeugenol acts primarily locally on sensory systems. All effects we discovered at the lowest dose required to anesthetize fish were merely on the visual and acoustic inputs into the neuron. Systemic effects on the neuron occur only at much higher concentrations of isoeugenol. The differences in dose-dependency and in time course of action of isoeugenol on the acoustic and the visual PSPs as well as the much higher sensitivity of the visual inputs allows two conclusions: First, isoeugenol does not exclusively and generally act on peripheral nerves that connect the sensory organs to the CNS. Second, it also does not act on the level of the central neuron itself. Rather, isoeugenol must act differently on each sensory organ and most likely includes an effect on the sensory organ itself. Here the effect on vision is particularly strong, however, followed by apparently full and quick recovery after anesthesia. In conclusion, isoeugenol seems to act mostly by blocking sensory inputs to the CNS. If an intervention includes strong acoustic or mechanosensory inputs, a much higher dose is required to also block these inputs. Our findings do exclude isoeugenol as an anesthetic agent for research on sensory biology. At low concentration, isoeugenol should only be used for noninvasive procedures, since the desired effect might be incomplete and highly depends on dose. Species-specific differences in dosing and many factors may influence anesthetic stage, and extrapolation is not straightforward. Nevertheless, isoeugenol, and perhaps other forms of eugenol, would be an interesting and efficient anesthetic agent in fish farming, but also in research laboratories where potentially stressful handling of fish requires anesthesia.

## Methods

### Experimental animals

We used *n* = 40 goldfish (*Carassius auratus* (Linnaeus, 1758)) to determine the effects of isoeugenol anesthesia in electrophysiological measurements (Supplementary Fig. 1a), and *n* = 20 additional fish in behavioral testing. In addition, we used data from *n* = 20 goldfish anesthetized with 2-phenoxyethanol. The experimental fish were taken from a pool of 120 fish obtained from a specialized retailer (Aquarium Glaser GmbH, Germany). At the time of the experiments, the fish had a standard length of 73 ± 1 mm and were sexually mature. We used both sexes in the present study, but did not consider sex as a factor, since the vital neuronal network containing the two MNs is present in both sexes and has the same significance for survival in both of them^29^. In addition, there has been – to our best knowledge – no study to date that shows sex differences in the MN network of goldfish. Before used in an experiment, the fish were kept in groups of up to 20 individuals per tank (250 ×50 x 50 cm) for at least 12 weeks. In these tanks, we used sand (grain size: 1–2 mm; 3–5 cm high) on the bottom, allowing the fish to dig. Light/dark photoperiod was 12:12 h. Water temperature was 20.0 ± 0.5 °C; water conductivity: 300 μS cm^−1^; pH 7.5; total hardness of water: 7.7°dH; NH_4_^+^ < 10 μg L^−1^; NO_2_^−^ < 5 μg L^−1^; NO_3_^−^ < 5 mg L^−1^. Water values were checked at least twice a week. Water changes (30%) were made once a week. Water of the same quality was used in experiments, but here the temperature was not allowed to vary by more than ± 0.1 °C. During the keeping period, the fish were fed daily (sera goldy (sera GmbH, Germany), defrosted mosquito larvae, floating fern (*Ceratopteris pteridoides*)) and examined for their general condition. Individuals with injuries or abnormal behavior were not used in any experiment. The selection of an experimental fish for one of the experiments occurred randomly from one of the keeping tanks. The persons selecting a fish for electrophysiological measurements did not know at the time of selection which anesthetic at which concentration would be used that day. They only made sure that the selected fish is in good general condition and responds to sensory stimuli, such as would be used for stimulation in the experiments subsequently. Fish that did not respond to the sensory stimuli were not used in the experiments. Other restrictions (e.g., sex) were not placed on the selection. The fish used in electrophysiological experiments were sacrificed after completing recording. The postmortem examination of the gonads revealed that all fish were sexually mature. We assume that this was also the case in the fish used in behavioral testing (which were not sacrificed after finishing testing). Animal care, surgical procedures and experiments were in accordance with all relevant guidelines of the German Animal Welfare Act and explicitly approved by the Council of State.

### Electrophysiological experiments

#### Anesthesia prior surgery and surgical procedure

Surgical intervention is required to access the MN for in vivo intracellular recording. Experimental fish therefore were taken from one of the keeping tanks and placed for 15 min in a small anesthetization tank containing either isoeugenol (CAS# 97-54-1; Sigma-Aldrich I17206; solved 1:10 in 95% ethanol) at the concentration of 10 mg L^−^^1^ or 2-phenoxyethanol (2-PE; CAS# 122-99-6; Sigma-Aldrich 77699) at the concentration of 400 mg L^−^^1^. These anesthetic concentration levels were chosen based on appropriate references to reach surgical (stage III.2)^41,42^ anesthesia^26,43,44^. After 10 min all experimental fish had ceased swimming and lost equilibrium. None of the fish showed any response to touch and to pressure exerted gently to the fish’s caudal peduncle. When this stimulation and subsequent handling yielded no response, the fish was placed in the electrophysiological recording chamber. Artificial respiration was established via a tube in the fish’s mouth. The aerated respiration water was delivered to the fish from a reservoir (respiration water tank) using a suitably adjusted pump (EHEIM universal 300; EHEIM GmbH, Germany; regular power: 300 L h^−1^, adjusted to 4.8 L h^−1^). To maintain anesthesia, the respiration water contained the same anesthetic in the same concentration as used for establishing anesthesia: either 10 mg L^−^^1^ isoeugenol or 400 mg L^−^^1^ 2-PE.

The two MNs are located in the medulla oblongata, which is part of the vertebrate hindbrain. To get access, we exposed the brain from above from optic tectum to vagal lobe. Note that none of the fish showed any response during surgical intervention, indicating the effectiveness of the stage III.2 anesthesia established either by isoeugenol [10 mg L^−^^1^] or by 2-PE [400 mg L^−^^1^]. The cerebellum was lifted upwards and cranially to expose the medulla. Meninges covering the medulla were removed. A piece of 2 mm of the spinal column was exposed in the area of the trunk. The large Mauthner axons run down the entire spinal cord. Electrical pulses applied to the spinal column therefore can be used to activate the MN antidromically. Activation of both MNs causes typical twitching of the experimental fish. This twitching was not ceased by either isoeugenol or 2-PE anesthesia^26^. After testing the correct positioning of the home-made bipolar stimulation electrode forwarding electrical pulses to the spinal column, we therefore injected d-tubocurarine (CAS# 6989-98-6; Sigma-Aldrich T2379; 1 μg g^−1^ body weight) to immobilize the fish for MN recording. After finishing measurements, the experimental fish were immediately sacrificed by mechanically destroying the brain.

### Experimental setup and procedure

In electrophysiological experiments, we used a bridge mode amplifier (BA-01X; npi electronic GmbH, Germany) in current-clamp mode. The reference electrode was positioned in muscle tissue. The sharp recording electrodes were made from 3 mm-glass capillaries (G-3; Narishige International Ltd., UK) and filled with 5 mol L^−^^1^ potassium acetate. The recording electrode was moved in the brain using a motorized micromanipulator (MP-285; Sutter Instrument, USA). We used established techniques to localize and to identify one of the two MNs for intracellular in vivo recording^21,30,37^. Recordings were filtered (Hum Bug Noise Eliminator; Quest Scientific, Canada) and digitized (A/D converter Micro1401; Cambridge Electronic Design Limited, UK) at 50 kHz. For further processing and analysis we used the acquisition software Spike2 (version 6; Cambridge Electronic Design Limited) and custom-made software written in Python.

After establishing stable MN recording, we started the presentation of our set of stimuli. A set of stimuli, as designed for the present study, contained repeated antidromic activation of the MN and repeated acoustic and visual stimulation of the fish. Each of the stereotyped stimuli was consecutively presented to the fish at least 40 times per set. We always presented the stimuli in the same order: first: antidromic stimulation; second: acoustic stimulation; third: visual stimulation (Supplementary Fig. 1b). Intervals between the stimuli were chosen in each case to avoid after effects and habituation. In total, presentation of a set of stimuli took about 10 min. Electrical pulses (10 µs in duration; inter-stimulus interval: 500 ms) used for antidromic MN activation were delivered by a constant-voltage isolated stimulator (DS2A2 – Mk.II; Digitimer Ltd., UK). Desired pulse amplitude was set just so to only elicit action potentials in the large MN axons, but not in any other axons that are thinner and would therefore require higher stimulation pulse amplitude. It was determined by first reducing pulse amplitude until antidromic stimulation did not activate the MN anymore. Then the pulse amplitude was increased slightly above threshold. For acoustic stimulation, we used an acoustical broadband pulse (duration: 1 ms; frequency distribution from 25 to 1000 Hz; peak amplitude at 300 Hz; sound pressure level (SPL): 145 dB re 1 μPa; inter-stimulus interval: 4 s) delivered by a multifunctional active loudspeaker (The box pro Achat 115 MA; Thomann GmbH, Germany). We measured SPL under water at the position of the fish in the recording chamber with a hydrophone (Type 8106; Brüel & Kjær, Denmark). For visual stimulation, we used a light-emitting diode (LED; RS Components GmbH, Germany), which was positioned directly in front of the ipsilateral eye (e.g., left MN, left eye). The emitted light flash had a duration of 7 ms. Inter-stimulus interval was 10 s. LED peak radiation at 569 nm was 700 μW m^−2^ nm^−1^ and the width at 100 μW m^−^^2^ nm^−^^1^ was 56 nm (range: 543–599 nm).

To test to which extent effects of isoeugenol depend on concentration and exposure period, the *n* = 40 experimental fish were randomly divided into four experimental groups (*n* = 10 fish each), which were all anesthetized with 10 mg L^−^^1^ isoeugenol. After establishing MN recording, we presented our set of stimuli to these fish for the first time (Supplementary Fig. 1a). Then, we increased the isoeugenol concentration from 10 either to 20, 40, or 60 mg L^−^^1^ by adding additional anesthetic agent to the respiration water tank. To quickly establish a uniform mixture, we used a circulation pump (EHEIM universal 600; power: 600 L h^−1^) in the respiration water tank. Ten minutes after finishing the first set presentation and after increasing isoeugenol concentration, we presented our set of stimuli a second time. Then, we again gave 10 min, before presenting our set of stimuli a third time, and so on (Supplementary Fig. 1a). All in all, we repeated the set presentation 5 times to the experimental fish after increasing isoeugenol concentration with an interval of 20 min (10 min to present the set of stimuli (Supplementary Fig. 1b), and 10 min between set presentations). In a fourth group of *n* = 10 fish we did not increase isoeugenol concentration, but proceeded in the same way. We used the data from this group to find out whether the long-lasting recording period itself affects the values we planned to evaluate in the present study. For our analyses, we were able to collect data from *n* = 6 (of 10) fish in which we did not increase isoeugenol concentration, from *n* = 7 (of 10) fish in which we increased concentration from 10 to 20 mg L^−^^1^, from *n* = 7 (of 10) fish in which we increased it to 40 mg L^−^^1^, and from *n* = 5 (of 10) fish in which we increased it to 60 mg L^−^^1^. The data of these in all *n* = 25 (of 40) fish were compared with data taken in *n* = 20 fish anesthetized with 2-PE [400 mg L^−^^1^]. In *n* = 1 experimental fish we failed to localize one of the MNs. In *n* = 14 experimental fish we failed to establish stable intracellular recording for at least 100 min (see Termination criteria).

### Rationale for the number of animals used

We used standard parameters for the biometric justification of animal numbers and set α to 0.05 and β to 0.2. Then, we used the following formula as recommended by the Karlsruhe Institute of Technology (KIT):

$$n\approx {\left(\left(2\left({z}_{1-\beta }+{z}_{1-\alpha }\right)s\right)/\left({m}_{c}-{m}_{e}\right)\right)}^{2}$$

Setting α to 0.05 results in z_1–α_ = 1.65. Setting β to 0.2 results in z_1–β_ = 0.84 (Values taken from the KIT IZMC handout); m_c_ = mean value of the control experiment; m_e_ = mean value of effect group; s = standard deviation of the higher mean value. Since the biorelevant effect size and standard deviation were unknown, we then had to make assumptions: Assuming a biorelevant effect size between 40 and 50% (in one direction or the other) and a standard deviation of about 20% of mean value (taken from experiences made in Machnik et al. (2018)^26^ and Schirmer et al. (2021)^27^), we determined the number of animals *n* ≈ 5. Due to the long-lasting recording period (and referring to the experiences made with long-lasting MN recordings in Machnik et al. (2018)^37^), we doubled this number to be able to obtain at least *n* ≈ 5 successful measurements.

### Termination criteria

Penetrating a neuron with a recording electrode to establish intracellular recording can affect the cell. After establishing intracellular recording, we therefore gave at least 10 min, before we started any measurement. In this time, we evaluated changes of the resting potential and did not start measuring until the resting potential was stable (changes of the resting potential of less than 2% for at least 5 min). When the resting potential did not stabilize, we attempted to establish stable intracellular recording in another position of the same MN or in the other one.

To ensure stable recording conditions after starting measuring and the presentation of our sets of stimuli, we further on continuously monitored the resting potential of the MN. When the intracellular recording was no longer stable (change in resting potential of more than 2% in less than 5 min) or broke off, we terminated the measuring and no further attempt was made to re-establish the intracellular recording at another recording site. Due to the repeated measures design of the analyses to determine effects in the isoeugenol anesthetized groups, data were only implemented in the analyses when at least 5 set presentations could be completed.

### Behavioral testing

We used *n* = 20 fish in behavioral testing. The fish were randomly divided in two experimental groups of *n* = 10 fish each. One group was exposed to isoeugenol [20 mg L^−^^1^] for 30 min. Then the fish of the exposure group were transferred to a tank containing fresh water and no anesthetic agent. After the transfer, the fish were observed for 30 min and a light flash as used in the electrophysiological experiments was presented at least once per minute. When a fish did not respond to a light flash, a second flash could be given within 30 to 60 s. Thereby, we evaluated when the fish started moving again, when they started swimming, and at what point they responded to the light flash stimulation again for the first time. In the control group, the fish were not exposed to isoeugenol, but also observed and visually stimulated.

Although it also may be of interest whether the reversibility of the effects on vision differs when higher doses are administered, we did not test higher concentrations: At higher concentrations breathing slows down too much in the experimental fish (with no artificial ventilation in behavioral testing), so the fish might suffer at the high concentrations over the extended exposure time. Because vision was blocked at much lower concentration, we used these lower concentrations to study if vision comes back.

### Statistics and reproducibility

Statistical analyses were run in the software Prism 8 (version 8.4.3 (471); GraphPad Software, USA) and performed two-tailed with α = 0.05. Averages are given as mean ± standard error of mean. *n* denotes the number of independent animal samples. When data from animals were pooled, we never used the measurement repetitions taken from the individual animals, but a single averaged value for each animal. To test whether data are distributed normally (Gaussian), we used the Shapiro-Wilk test. When data were normally distributed, we used a parametric test design, otherwise a non-parametric one. To determine whether measurements collected in isoeugenol [10 mg L^−^^1^] anesthetized fish differ from that collected in 2-PE fish, we used an unpaired t-test or a Mann-Whitney test, respectively. To determine whether there are additional effects of higher isoeugenol concentration (≥ 20 mg L^−^^1^) at all in comparison to the pre-increase (= 10 mg L^−^^1^) state, we used the paired t test or the Wilcoxon test, respectively: Here, we compared the values measured before increasing isoeugenol concentration with those measured 90 min after the increase. To determine the exposure time an effect needed to occur after increasing isoeugenol concentration, we used the mixed-effects analysis with Geisser-Greenhouse correction. As post-hoc test we used the Dunnett test.

### Reporting summary

Further information on research design is available in the Nature Portfolio Reporting Summary linked to this article.

## Supplementary information

Supplementary Information

Description of Additional Supplementary Files

Supplementary Data

Reporting Summary

## Data Availability

The datasets generated and/or analyzed are available from the corresponding author on reasonable request. The source data for the graphs and charts in the figures are present in the supplementary data file.
