# Supplementary material for: Recordings in an integrating central neuron reveal the mode of action of isoeugenol
Source: Commun Biol. 2023 Mar 23;6:309. doi: 10.1038/s42003-023-04695-4 (PMC10036640; doi:10.1038/s42003-023-04695-4)
Supplement: Supplementary file 1 — Supplementary Information [file 42003_2023_4695_MOESM1_ESM.pdf]

Supplementary Figure 1. Overview of the experimental design.

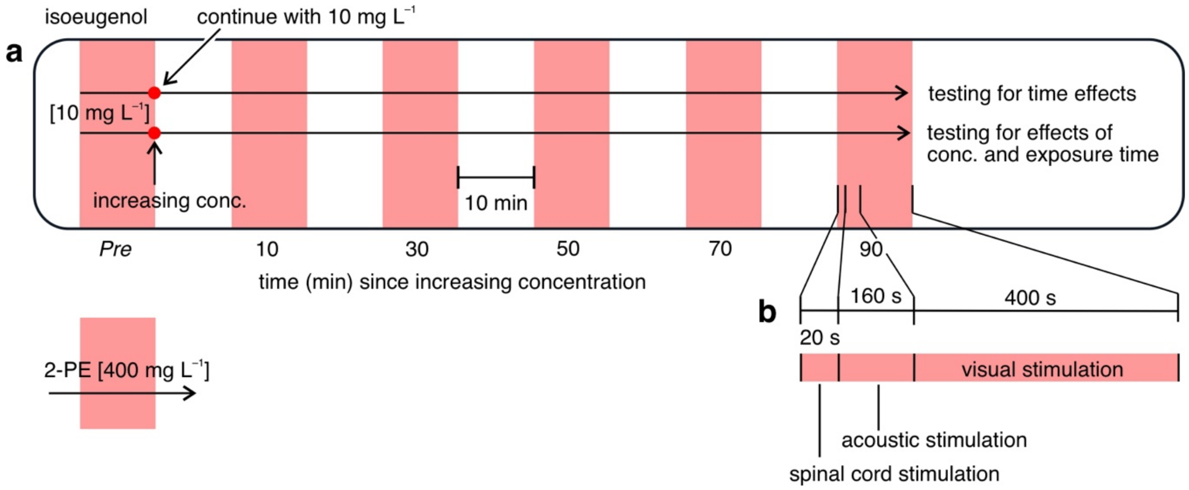

(a) Illustration of timing during the experiment and how respective (sub-)groups were treated. When isoeugenol concentration was increased, it was increased from 10 mg L^{-1} to either 20, 40 or 60 mg L^{-1} at the indicated time point. Periods of set presentations are marked by red rectangles. The presentation of a set of stimuli took about 10 min. Between set presentations 10 min were given. (b) A set of presentations contained repeated antidromic activation of the Mauthner neuron, followed by repeated acoustic and then visual stimulation of the fish.

Supplementary Figure 2. Time course of delay, amplitude, integrated amplitude I_1 and slope of the acoustically induced PSPs in the Mauthner neuron.

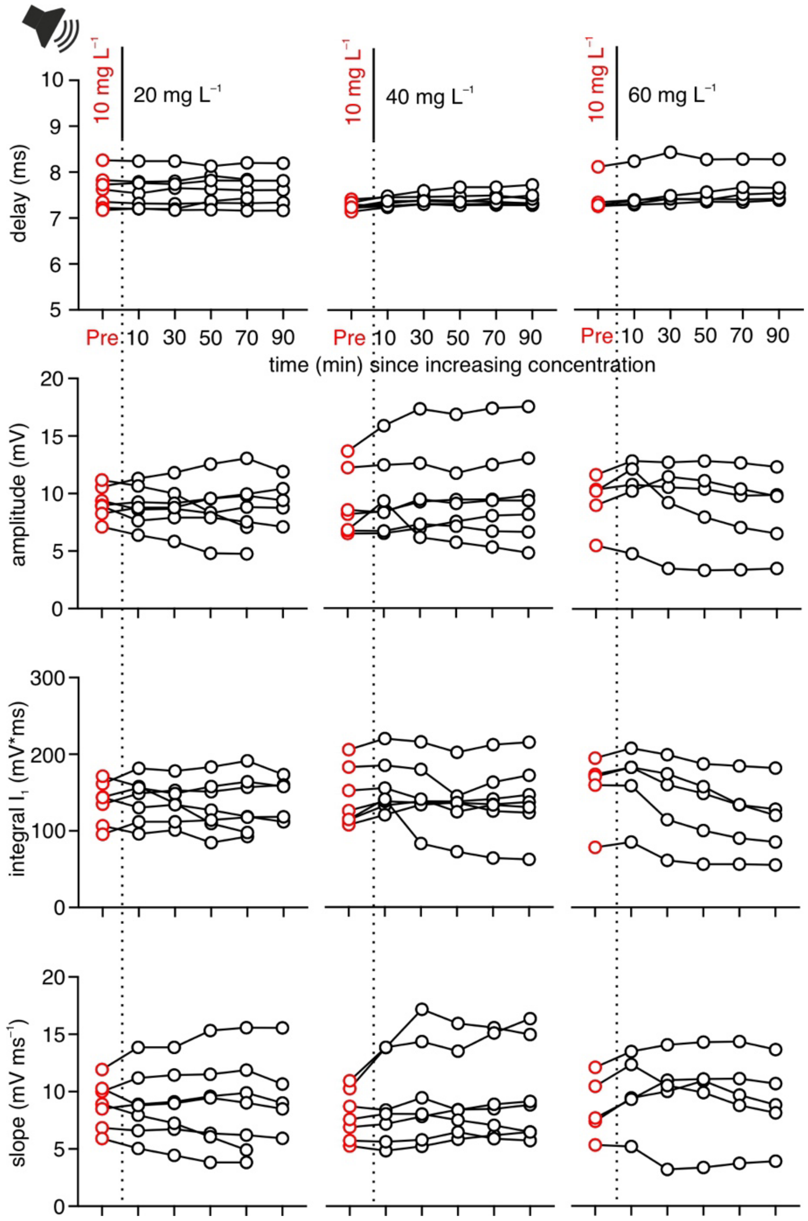

After an increase in isoeugenol concentration at time = 0 min (dotted vertical lines) from 10 to either 20, 40 or 60 mg L^{-1} no significant deviations from baseline levels (Pre: 10 mg L^{-1}; red circles) occurred, at any concentration, on delay, amplitude, I_1 and slope. Connected circles indicate mean values of individual fish.

Supplementary Figure 3. Controls to show that the late effects occur due to the change in isoeugenol concentration and not due to a decline in the recording quality.

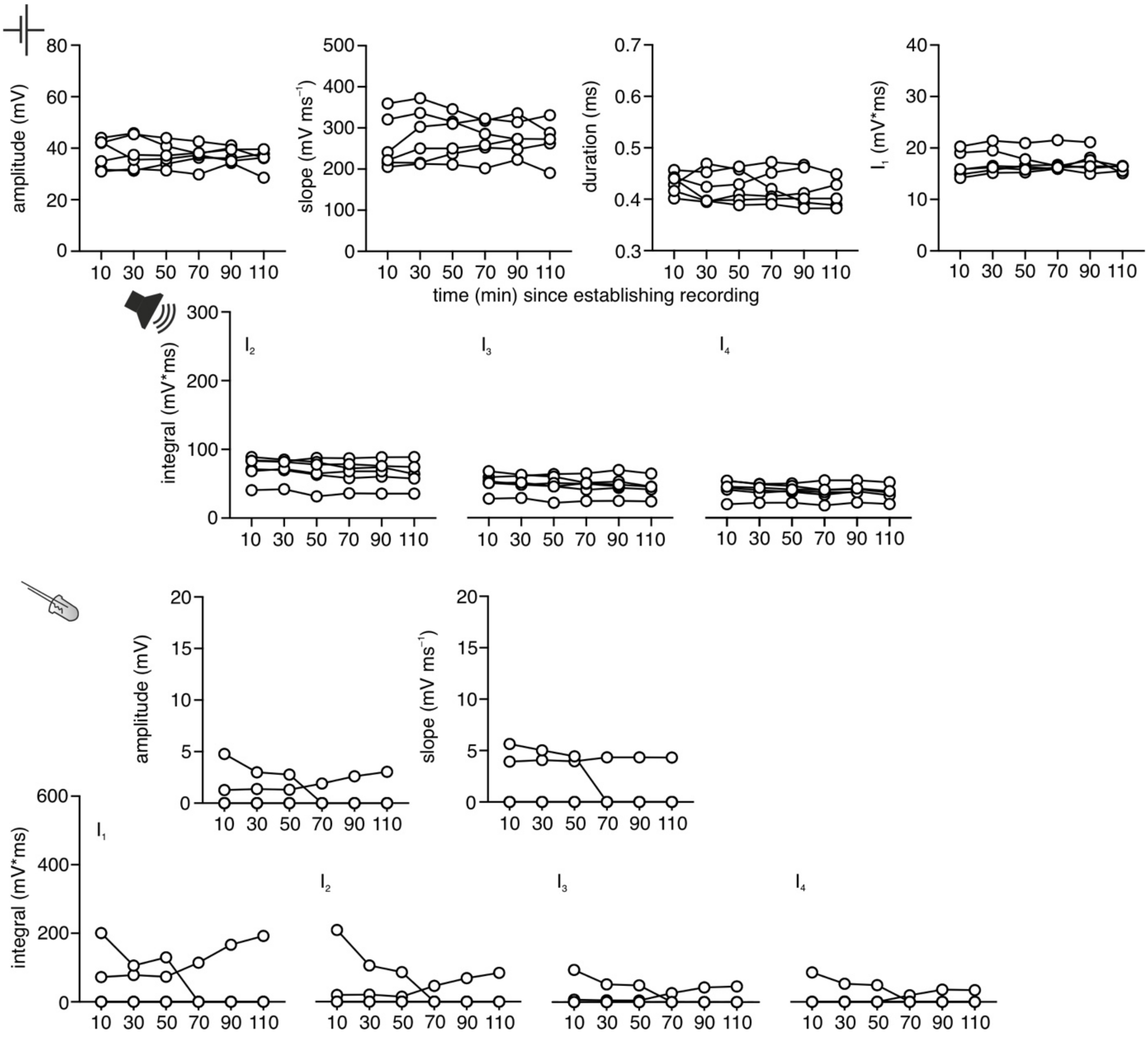

For the values that changed significantly after an increase in isoeugenol concentration, we checked whether time effects (and not the dose effects) could be responsible for the observed changes. During intracellular recording changes in the evaluated values might occur due to the continuous leakage of ions from the recording electrode or from damaging the membrane of the penetrated cell. This would also result in changes over time. However, in the control fish that always remained at the initial low isoeugenol concentration (Suppl. Fig. 1a), with all other recording and testing conditions equal, none of the effects observed after increasing isoeugenol concentration occurred. Hence, significant dose-dependent changes were due to increased concentration but not due to deterioration of the cells or of the recordings. Connected circles indicate mean values of individual fish.

Supplementary Figure 4. A counterexample against the systemic action of isoeugenol.

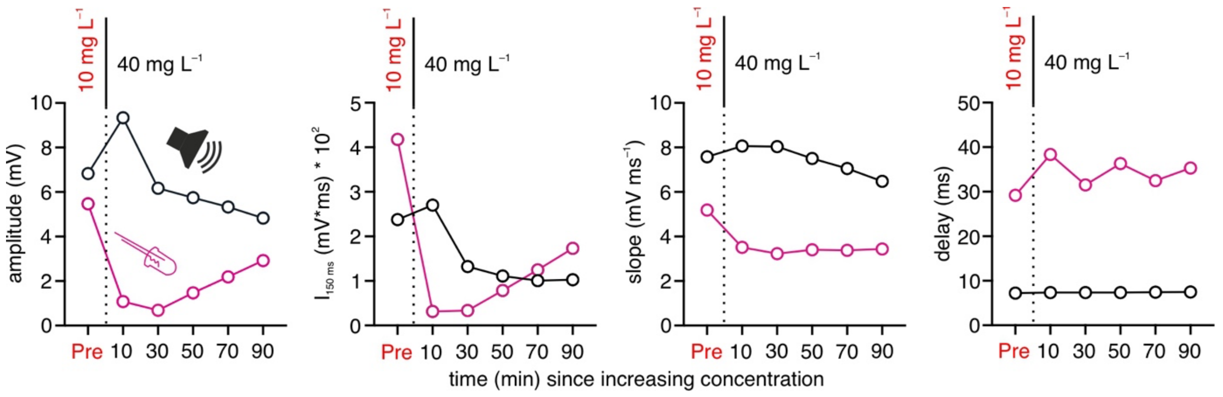

Time course of action of isoeugenol anesthesia on hearing and vision in one of the experimental fish. Increasing isoeugenol concentration from 10 (= Pre) to 40 mg L^{-1} had an opposite effect on acoustic and visual PSPs. In the period from 30 to 90 min of exposure to the higher dose PSP amplitude, the area under the first 150 ms of the PSP (acoustic = I_1 to I_3; visual = I_1 to I_2) and the maximal slope of the visually induced PSP increased (linear regression analysis: goodness of fit: $R^2 \geq 0.7164$; regression lines all with positive slope), but decreased for the acoustically induced PSP (linear regression analysis: goodness of fit: $R^2 \geq 0.7841$; regression lines all with negative slope). Delay was not affected for both the visually and the acoustically induced PSP. This finding could not be explained if isoeugenol acted generally on central neuron function or on peripheral nerves forwarding the information to the CNS.
