# Supplementary material for: Recordings in an integrating central neuron reveal the mode of action of isoeugenol
Source: Commun Biol. 2023 Mar 23;6:309. doi: 10.1038/s42003-023-04695-4 (PMC10036640; doi:10.1038/s42003-023-04695-4)
Supplement: Supplementary file 2 — Description of Additional Supplementary Files [file 42003_2023_4695_MOESM2_ESM.pdf]

Description of Additional Supplementary Files

File name: Supplementary Data
Description: The source data for the graphs and charts in the figures.
